# Distal Radial Artery Access: The Future of Cardiovascular Intervention

**DOI:** 10.7759/cureus.7201

**Published:** 2020-03-07

**Authors:** Zaid Nairoukh, Saira Jahangir, Dennis Adjepong, Bilal Haider Malik

**Affiliations:** 1 Internal Medicine, California Institute of Behavioral Neurosciences and Psychology, Fairfield, USA; 2 Neuroscience, California Institute of Behavioral Neurosciences and Psychology, Fairfield, USA; 3 Neurological Surgery, California Institute of Behavioral Neurosciences and Psychology, Fairfield, USA

**Keywords:** distal radial artery access, distal radial artery approach, snuffbox approach, snuffbox access, cardiovascular intervention, coronary angiography, coronary intervention, coronary angioplasty

## Abstract

Access sites for coronary intervention have been changing over the last several decades, from the femoral artery to the radial artery and then to the distal radial artery. Distal radial access, which was first used in 2017 and is still not recommended by the guidelines, shows a higher success rate and less complications than other sites; therefore, it might be the future for cardiovascular intervention. In this study, we reviewed almost all of the articles that are related to the distal radial access, from 2017 to present, and summarized the technique, success rate, advantages, disadvantages, and noncardiac use of this access site.

## Introduction and background

Over the last several years, many changes have occurred in the treatment of ischemic heart diseases; one of these changes is the route of arterial access for both coronary angiography and percutaneous coronary interventions.

Access changed from transfemoral to transradial approach (TRA), as it has less bleeding complications, decreased hospital mortality rate, less access site complications, and is cost-effective as compared to the transfemoral approach [1]. In 2015, the European Society of Cardiology guidelines for the management of acute coronary syndrome gave class I recommendation to use the TRA as the preferred method of access for any percutaneous coronary intervention irrespective of clinical presentation [2].

However, the use of TRA is not free of limitations; many complications have been associated with the TRA due to small diameter, such as radial artery occlusion (RAO) (The reported incidence of RAO is highly variable in the range of 2%-10%, and a meta-analysis by Rashid showed that the RAO incidence within 24 hours was 7.7%), radial artery spasm, radial arterial perforation, radial artery pseudoaneurysm, arteriovenous fistula, bleeding, nerve damage, and complex regional pain syndrome [3,4].

Recently, a new approach was proposed to overcome these limitations and also to give the advantage over the transfemoral approach; this was a “distal transradial approach (dTRA) (snuffbox approach)”. The first publication was proposed in 2017 by Kiemeneij, and after that, a large number of studies have evaluated the safety and feasibility of this new approach [5].

In this study, we review the most related and recent articles to know more about the dTRA through rapid revision of radial artery anatomy. We then discuss preparation and technique of this approach and how proper preparation can improve success rate, and compare advantages and disadvantages of this approach over the old approach. We also touch on other noncardiac interventions that might be done by this approach. Finally, we attempt to confirm if this approach could be the future guideline for cardiovascular intervention.

## Review

Anatomy

The anatomical snuffbox is defined as a triangular depression on the dorsum of the hand at the base of the thumb; it is bordered by the abductor pollicis longus and extensor pollicis brevis laterally and the extensor pollicis longus medially. The floor is formed by the scaphoid and trapezium carpal bones. The contents of the anatomical snuffbox include the distal radial artery (DRA), cephalic vein, and superficial branches of the radial nerve. There are two sites at which the radial pulse can be found: in the anatomic snuffbox and the first intermetacarpal space; these two sites represent alternative puncture points for TRA [5]. As the radial artery has reached the anatomic snuffbox, it has already given rise to some branches that, in case of vessel occlusion, could avoid flow interruption. Flow interruption appears to play a significant role in artery occlusion. Indeed, in a retrospective series, the absence of blood flow during the hemostasis process significantly increased the risk for RAO [6]. On this background, dTRA could maintain forearm radial artery patency during hemostatic compression or in case of occlusion at the puncture site [7].

Diameter

It is essential to know that DRA diameter is 80% less than the proximal, and this may affect suitable French sheath that can be used and it varies according to sex, race, and other factors. The diameter of the DRA in females tends to be smaller than that in males. Some studies show that hypertensive patients had larger radial artery diameter than normal. Other studies show that the diameter of DRA positively correlated with both body weight and basal metabolic index. Ulnar arteries were slightly larger than radial arteries and may be used as an alternative to DRA [8-10].

Here are some examples of means of internal diameter of radial artery from different areas: in Korea (2.57 ± 0.50 mm), in India (2.325 ± 0.4 mm), in Japan (2.6 ± 0.5 mm), in Pakistan (2.25 ± 0.4 mm), in Singapore (2.45 ± 0.54 mm), in Turkey (2.05 ± 0.34 mm) [8-13].

Ultrasound guidance

A blind puncture increases the risk of tendon damage. And at the same time, the double-wall technique can irritate the underlying periosteum and increase the risk of hematoma formation. The utilization of the ultrasound (US) allows identification of anatomical landmarks, and enables accurate vessel access, especially in impalpable DRA. The probe can be used to perform a compressibility test to confirm that the target vessel corresponds to the radial artery rather than the cephalic vein. Accurate scanning can identify the superficial branch of the radial nerve, thus avoiding potential injury. A further benefit of US guidance is that the operator can measure the vessel size before puncturing. The outer diameters of 5 French, 6 French, and 7 French introducer sheaths are usually 2.3, 2.6, and 2.8 mm respectively, and it can help to determine whether the radial artery can accommodate the required procedural sheath and hardware, and to choose a smaller diameter sheath in order to reduce the risk of vascular injury, unnecessary patient pain, and RAO [10,14].

Technique

The left upper arm is placed comfortably on a cushion on the left side of the patient. The left hand is bent over towards the patient's right groin. After disinfection, the patient is covered with a sterile drape. To bring the DRA to the surface of the radial fossa, the patient grasps his thumb under the other four fingers. After subcutaneous injection of 3-5 cc xylocaine, the artery is punctured, preferably with a 21 gauge open needle, under an angle of 30-45 degrees and from lateral to medial. The needle directed to the point of most forceful pulse, proximal in the anatomical snuffbox. A through-and-through puncture is not recommended because the contact of the needle to the periosteum of scaphoid and trapezium bones can be painful. After successful puncture, a flexible, soft, J-shaped 0.21" metallic wire was inserted. After administration of a spasmolytic cocktail (200 mcg of nitroglycerine and 5 mg of verapamil) and a weight-adjusted dose of heparin, the operator can take up a position at the level of the patient’s knees to manipulate the 0.35” wire, the catheters, and the intracoronary devices. After the procedure, the sheath is pulled out for 3 cm, after which a SafeGuard (Merit Medical Systems,South Jordan, UT) hemostasis band is placed over the puncture site. At inflation of 3 mL of air into the air compartment, the sheath is pulled out completely, followed by an extra injection of 2 mL of air. This band is left in situ for two or maximally three hours, after which deflation of air is started and completed within half an hour. Alternatively, a small pile of gauze is placed over the puncture site during sheath removal, followed by the application of a semi-elastic bandage, which is left in situ for two to three hours [5].

Success rate

Multiple studies have been publishing over the last three years, and most of them have a high success rate (approximately 90%). Table 1 shows the success rate and complications of distal radial artery access in approximately all studies after Kiemeneij. 

**Table 1:** Success rate and complication of distal radial artery access in cardiovascular intervention. PCI, percutaneous coronary intervention; dRAO, distal radial artery obstruction; pRAO, proximal radial artery occlusion

Year	Author	Country of the study	Number of patients (PCI)	Success rate, %	Complications, %
May 2017	Kiemeneij [5]	Netherlands	70 (25)	89	dRAO: 1.5%
Jul 2017	Amin et al. [15]	Bangladesh	50	98	No complications observed
Jan 2018	Roghani-Dehkordi et al. [16]	Iran	235	94	No complications observed
Mar 2018	Al-Azizi and Lotfi [17]	US	22 (7)	100	No complications observed
Mar 2018	Valsecchi et al. [18]	Italy	52 (25)	90	pRAO: 4%, artery spasm: 2%
Mar 2018	Soydan and Akin [19]	Turkey	54 (20)	96.3	Artery spasm: 3.7%
Aug 2018	Coughlan et al. [20]	Ireland	47	100	No complications observed
Aug 2018	Kim et al. [21]	Korea	150 (42)	88	Hematoma: 4.9%
Sep 2018	Koutouzis et al. [22]	Greece	100	70	No complications observed
Oct 2018	Ziakas et al. [23]	Greece	49 (8)	90	Hematoma: 16%, bleeding: 4.5%, artery spasm: 16%, pain: 9%,
Dec 2018	Wretowski et al. [24]	Poland	218 (48)	89.4	No complications observed
Feb 2019	Aoi et al. [25]	US	202 (90)	99.5	Hematoma: 10.1%, dRAO: 1.0%, arteriovenous fistula: 0.5%
Mar 2019	Maitra et al. [26]	India	55	87.3	Hematoma 2%
Apr 2019	Norimatsu et al. [10]	Japan	74 (27)	91	No complications observed
May 2019	Gasparini et al. [27]	Italy	41(41)	82.9	Artery spasm: 10%, dRAO: 2.4%
May 2019	Mizuguchi et al. [28]	Japan	228 (77)	99.5	Hematoma: 4.4%, pRAO: 0.4%, dRAO: 3.1%, numbness: 0.9%
Jul 2019	Vefali et al. [13]	Turkey	102 (24)	95.1	No complications observed
Sep 2019	Lee et al. [29]	Korea	200 (86)	95.5	Hematoma: 7.4%, arterial dissection: 0.5%, numbness: 1%
Dec 2019	Aqel et al. [30]	Palestine	200 (17)	98	No complications observed

Advantage

dTRA technique seems to have more advantages. First, the arm position during the intervention is comfortable for the patient, who does not have to expose the palmar side of the arm while flexing the upper arm towards the operator. No equipment or investments are necessary to support the patient's left arm. The operator can work as usual from the right side of the patient and does not need to bend over the patient to reach for the left radial artery. Second, there is low rate of DRA obstruction Since antegrade flow through the superficial palmar arch is still maintained, the radial artery does not thrombose in case of occlusion of the radial artery in the snuffbox. Other advantages include early hemostasis, low risk for hematoma formation, low level of pain perceived by patients, reduced risk of compartment syndrome, saving the radial artery for possible future coronary artery bypass graft, and the ability of the operator to work at a safe distance from the radiation source [5,13,22,31]. Finally, it might be a potential site for retrograde recanalization of RAO [32].

Disadvantage

The disadvantages of dTRA are that they are technically more demanding and time-consuming, especially in access time. The radiation time was more. The snuffbox radial artery is smaller in diameter than the radial artery, and there is a higher risk of puncture-mediated vasospasm than TRA. However, in DRA vasospasm, we can cross to multiple other accesses like distal ulnar or contralateral dTRA, proximal TRA, or transfemoral. Lastly, the short length of a typical radial catheter is a significant drawback to the snuffbox technique. Given that the snuffbox artery is 5 cm below the common radial entry site, these catheters may, therefore, be too short, especially in taller patients [13].

Noncardiac use

Distal radial access can also be used for interventions other than cardiac, and a study in 2017 demonstrates using this approach in 50 visceral interventions [33]. Another case series study in 2019 on 85 patients demonstrates the feasibility of this approach as diagnostic in cerebral angiography [34]. Distal radial access is used for proximal RAO even if it associated with acute coronary syndrome, based on a case reported recently [33]. The last one, which is common, is using the DRA for arteriovenous fistula in dialysis, which affords more advantages than the traditional proximal fistula [35,36].

## Conclusions

Distal radial access is a new site for cardiovascular interventions, and it has several advantages over the old access sites. The main advantages are less arterial obstruction and short hemostasis. The main disadvantage is the difficulty in cannulation. However, more studies, especially randomized studies and meta-analyses, are needed to be a guideline in the future.
